# Prediction of Cancer Drug Resistance and Implications for Personalized Medicine

**DOI:** 10.3389/fonc.2015.00282

**Published:** 2015-12-17

**Authors:** Manfred Volm, Thomas Efferth

**Affiliations:** ^1^Faculty of Medicine, Ruprecht Karls University, Heidelberg, Germany; ^2^Department of Pharmaceutical Biology, Johannes Gutenberg University, Mainz, Germany

**Keywords:** chemotherapy, drug resistance, individualized therapy, survival times

## Abstract

Drug resistance still impedes successful cancer chemotherapy. A major goal of early concepts in individualized therapy was to develop *in vitro* tests to predict tumors’ drug responsiveness. We have developed an *in vitro* short-term test based on nucleic acid precursor incorporation to determine clinical drug resistance. This test detects inherent and acquired resistance *in vitro* and transplantable syngeneic and xenografted tumors *in vivo*. In several clinical trials, clinical resistance was predictable with more than 90% accuracy, while drug sensitivity was detected with less accuracy (~60%). Remarkably, clinical cross-resistance to numerous drugs (multidrug resistance, broad spectrum resistance) was detectable by a single compound, doxorubicin, due to its multifactorial modes of action. The results of this predictive test were in good agreement with predictive assays of other authors. As no predictive test has been established as yet for clinical diagnostics, the identification of sensitive drugs may not reach sufficiently high reliability for clinical routine. A meta-analysis of the literature published during the past four decades considering test results of more than 15,000 tumor patients unambiguously demonstrated that, in the majority of studies, resistance was correctly predicted with an accuracy between 80 and 100%, while drug sensitivity could only be predicted with an accuracy of 50–80%. This synopsis of the published literature impressively illustrates that prediction of drug resistance could be validated. The determination of drug resistance was reliable independent of tumor type, test assay, and drug used in these *in vitro* tests. By contrast, chemosensitivity could not be predicted with high reliability. Therefore, we propose a rethinking of the “chemosensitivity” concept. Instead, predictive *in vitro* tests may reliably identify drug-resistant tumors. The clinical consequence imply to subject resistant tumors not to chemotherapy, but to other new treatment options, such as antibody therapy, adoptive immune therapy, hyperthermia, gene therapy, etc. The high accuracy to predict resistant tumors may be exploited to develop new strategies for individualized cancer therapy. This new concept bears the potential of a revival of predictive tests for personalized medicine.

## Introduction

Chemotherapy of malignant tumors is essentially based on the results of prospective, randomized, double-blind phase III studies and corresponding clinical guidelines. However, the clinical response of the individual patient still remains uncertain, although the statistical probability of treatment success is known within large groups of patients from clinical studies. Tumors differ in their molecular architecture and biological behavior from patient to patient and even within the same tumor. There is a large heterogeneity between different subpopulations of tumor cells.

Drug resistance is a major reason for failure of cancer chemotherapy. In present clinical practice, drug resistance can only be recognized during larger periods of treatment. It, therefore, would be of great value for each individual patient to determine resistance before commencing treatment with antineoplastic substances. In nearly 50% of all cancer cases, resistance to chemotherapy already exists before drug treatment (1). Meanwhile, the knowledge of various resistance mechanisms has increased over the years (2, 3). While the responsiveness of tumor cells to targeted anti-cancer drugs (e.g., HER2- or estrogen-receptor-targeting small molecules) can be predicted by pre-therapeutic determination of their corresponding targets, the situation is more complicated for clinically long established cytotoxic drugs, where the molecular targets are either less well-defined or which have broader modes of action. Here, diagnostic tests are desirable to predict the response of tumors to treatment. If a tumor is resistant, therapy might only give rise to toxic effects in various normal tissues without having any major influence on tumor growth (4). The question therefore arises, whether the general clinical recommendations are optimal or whether they need to be improved by resistance testing.

Many different methods to assay sensitivity or resistance of tumors to chemotherapy have been developed over the last decades (5, 6). We used a test in which a cell suspension is prepared from fresh tumor biopsies. The cells are incubated over 3 h with cytostatic test drugs and the uptake of radioactive nucleic acid precursors (^3^H-thymidine or ^3^H-uridine) is determined during the last hour of incubation (7, 8). The important advantages of our test procedure are (1) its simplicity, (2) that the cells do not need to be long-term cultured before testing, and (3) that practically all tumor types can be tested. This review gives a synopsis of this test system.

### Detection of Acquired Resistance

**Figure 1:**
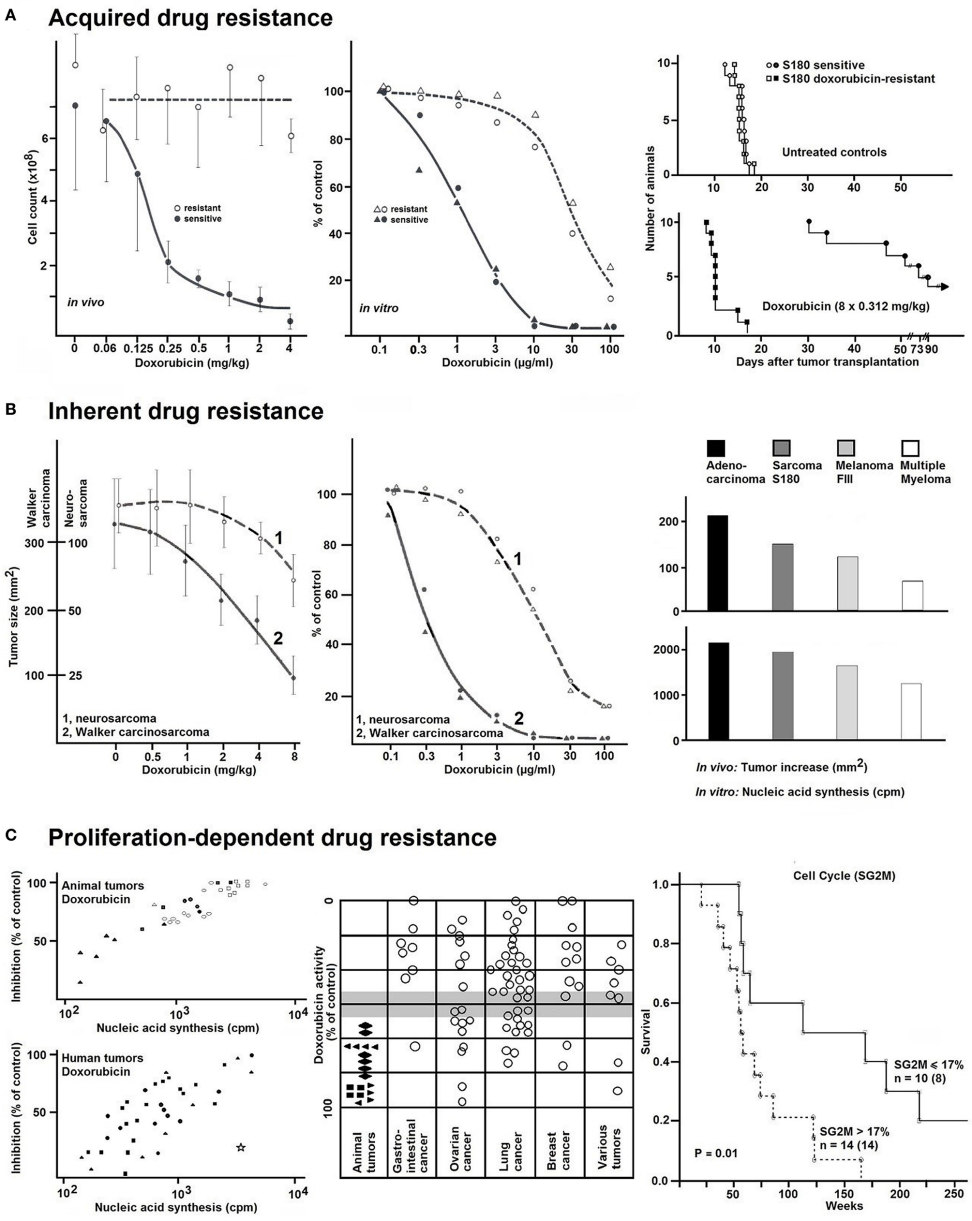
**(A)** The effects of different doxorubicin concentrations on doxorubicin-resistant or doxorubicin-sensitive ascites tumor cells of murine sarcoma 180 *in vivo* (left). Resistant tumor cells grown in mice were treated with doxorubicin (3 × 0.5 mg/kg BW per week) during 25 passages. The cytotoxic effect was measured by determination of the cell count. Average values ± SD are from seven tumors at each point. Corresponding results (middle) using the *in vitro* short-term test. After incubation of the tumor cells with different concentration of doxorubicin for 2 h, radioactive nucleic precursors (^3^H-uridine) were added for another hour. The non-incorporated radioactivity was extracted and the incorporated radioactivity determined by liquid scintillation counting. Uptake values were expressed as percentages of controls. Right: survival curves of mice bearing sensitive or resistant sarcoma S180 cells without or with doxorubicin treatment. Without therapy, the survival times for the animal with sensitive or resistant tumors were the same. With therapy, the survival times of both groups were significantly different. *n* = 60 mice. Data were taken from Ref. (9). **(B)** The effect of different concentrations of doxorubicin in slowly growing (1 = neurosarcoma) and rapidly growing (2 = Walker carcinosarcoma) animal tumors. Left: tumor size under therapy (square millimeter). Average values ± SD were from seven tumors at each point (*n* = 84 rats). Middle: ^3^H-uridine incorporation *in vitro*. Values (% of controls) were the averages from two tumors with duplicate determinations. Data were taken from Ref. (7). Right: relationship between tumor growth and cytostatic activity in various transplantation tumors (adenocarcinoma, sarcoma S180, melanoma FIII, and multiple myeloma) grown in different species (mouse, rat, and hamster). Right, top: tumor increase *in vivo* within 1 day (square millimeter). Right, bottom: ^3^H-Thymidine incorporation *in vitro* (cpm). Data were taken from Ref. (7). **(C)** The proliferation-dependent drug resistance in animal and human tumors. The variable tumor response to doxorubicin *in vitro* was assayed with a fixed concentration of 10^−2^ mg/ml (left and middle). Right: survival curves of patients with ovarian carcinomas subdivided according to the cell cycles phases (proportion of SG2M-phases ≤ or >17%). Flow cytometric analyses were carried out using an ICP-22 (PHYWE AG, Göttingen, Germany). For measurements of DNA content, a mixture of propidiumiodide and 4–6-diamidino-2-phenylindole was simultaneously applied with RNAse after methanol fixation and protease digestion. Data were taken from Ref. (11).

The usefulness of any resistance test depends on the degree, to which *in vitro* results are correlated with clinical results. To prove an *in vitro* short-term test to detect drug resistance, we generated different tumor lines, which were resistant toward doxorubicin, daunorubicin, cytosine-arabinoside, or cyclophosphamide (9, 10). As exemplarily shown in Figure 1A, sensitive and doxorubicin-resistant sarcoma 180 ascites tumor cells grown in mice were used. After treatment with doxorubicin over 25 passages, resistance to this drug was developed in animals (Figure 1A, left). This doxorubicin resistance was also detectable using this *in vitro* short-term test (Figure 1A, middle). Upon doxorubicin treatment, mice bearing resistant (pre-treated) tumor cells revealed significantly shorter survival times than mice with non-pre-treated tumor cells.

In addition to determination of resistance at a given time point, it was also possible to detect gradual increase or decrease during the development or reversion of resistance in tumor lines (12).

### Detection of Inherent Resistance

Walker carcinosarcoma and neurosarcoma both grown subcutaneously as solid tumors in rats provide suitable models as rapidly and slowly growing tumors, respectively. If left untreated, rats bearing Walker carcinosarcoma survived for 10 days and those bearing neurosarcoma for 10 weeks. The tumors responded to drug treatment in a growth rate-dependent manner. For example, doxorubicin had only weak effects on neurosarcoma, whereas the growth of Walker carcinosarcoma was appreciably inhibited by the same concentrations of doxorubicin (Figure 1B, left). This different proliferation-dependent sensitivity was also observed in the *in vitro* short-term test (Figure 1B, middle). We have obtained similar results with other transplantation tumors (adenocarcinoma, sarcoma S180, melanoma FIII, and multiple myeloma) grown in different species (mouse, rat, and hamster) (Figure 1B, right) (7).

The results obtained in few transplantation tumors were confirmed in large panels of animal and human carcinomas. Some carcinomas were very strongly affected by doxorubicin, whereas others showed no or only moderate effects. This variable tumor response to doxorubicin was correlated with the proliferation rate of these tumors (Figure 1C, left). A comparison between animal transplantation tumors and clinical human tumor specimens showed that animal tumors tend to be more sensitive than human ones (Figure 1C, middle). In general, tumors with high incorporation rates of nucleic acid precursors showed more pronounced inhibitory effects and *vice versa* (13).

To explore the relevance of proliferation-dependent drug response for patient survival, we investigated fresh surgical specimens of previously untreated ovarian carcinomas (Figure 1C, right) (11). All patients underwent surgery and subsequent chemotherapy, and all patients had a minimum of 5 years of follow up. Patients with highly proliferative tumors (proportion of SG2M-phase cells >17% as measured by flow cytometry) had shorter survival times than those with low proliferating tumors (proportion of SG2M-phase cells ≤17%) (*p* = 0.01). Similar results were obtained with lung carcinomas (14). This is in agreement with the general clinical observation that cancer chemotherapy is most successful, if applied for rapidly growing malignant cells (Figure 1C, right) (13).

## Clinical Studies

Survival curves differed, if patients were distributed into two groups on the basis of the *in vitro* short-term test with doxorubicin. Patients with *in vitro* resistant tumors died sooner than *in vitro* sensitive ones. Lung cancer patients, who refused chemotherapy lived on average only as long as patients with *in vitro* resistant tumors (8).

**Figure 2:**
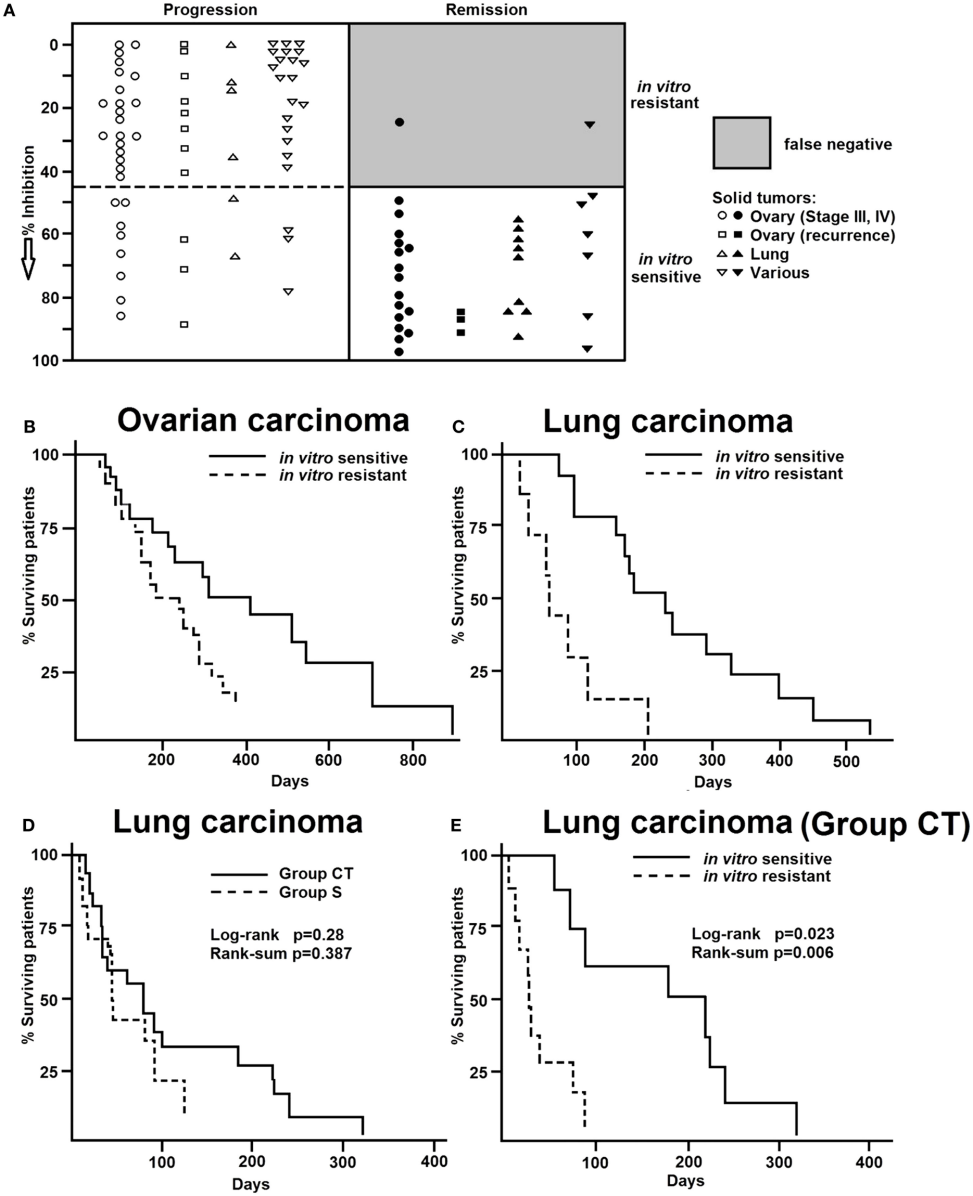
**(A)** Comparison of results of the *in vitro* short-term test and clinical chemotherapy of human tumors. Values represent the inhibition (%) of ^3^H-uridine incorporation. Closed symbols, tumors responsive to clinical chemotherapy. Open symbols, Tumors non-responsive to clinical chemotherapy. Data were taken from Ref. (15). Overall survival curves of patients with **(B)** ovarian carcinoma or **(C)** lung carcinoma separated into either resistant or sensitive groups according to the *in vitro* short-term test results. Data were taken from Ref. (15). **(D)** Of the 32 patients with previously untreated adenocarcinoma of the lung (stage III), 14 were treated with surgery alone (group S) and 18 were treated with surgery plus chemotherapy (group CT). The survival curves were not different between the S and CT groups (log-rank *p* = 0.63, rank-sum *p* = 0.39). **(E)** However, when the same data were analyzed on the basis of the *in vitro* short-term test, a different pattern appeared. CT patients with *in vitro* sensitive tumors were lived significantly longer than those with resistant tumors (log-rank *p* = 0.023, rank-sum *p* = 0.006). Data were taken from Ref. (16).

Results of these clinical pilot studies encouraged us to start a controlled clinical trial predominantly in ovarian and lung cancer, but also in other tumor types (Figure 2). In a multi-centric trial conducted by nine different hospitals, results obtained by the *in vitro* short-term test were compared with the clinical response of patients (15). Seventy-two patients with ovarian carcinoma, 24 patients with lung carcinoma, and 18 patients with various other tumor types were treated according to standardized therapy schedules (5-fluorouracil and cyclophosphamide for ovary carcinoma, cyclophosphamide, methotrexate, 5-fluorouracil for lung cancer) (Figure 2A). The remaining patients received different therapy regimens. Using the *in vitro* short-term test, dose–response curves were generated for doxorubicin as well as 5-fluorouracil and 4-OOH-cyclophosphamide (the *in vitro* active metabolite of cyclophosphamide). Remarkably, doxorubicin was most accurate compound compared to the other two drugs to predict clinical responsiveness to chemotherapy (Figure 2A) independent of the specific therapy regimen.

Summing up the results of all tumors studied in this trial, we have 151 matched comparisons of *in vitro* test results and clinical response rates to chemotherapy (Figure 2A). Of them, 76 had been classified as resistant and 75 as sensitive by the *in vitro* short-term test. Of the 76 tumors resistant *in vitro*, 56 were clinically progressive (73%), two were in remission and 19 (25%) showed no change. The 75 *in vitro* sensitive tumors showed the following clinical courses: 18 (24%) were progressive, 40 (53%) were in remission, and 17 (23%) were unchanged. If only strict clinical criteria (progression or remission) were applied and compared to the *in vitro* test results, 55 of the 57 *in vitro* resistant tumors were clinically progressive (96%) and only 40 of 58 *in vitro* sensitive tumors reached clinical remission (69%) (Figure 2A). Thus, drug resistance was predictable with high accuracy, but not drug sensitivity. Furthermore, drug resistance has been detected *in vitro* by doxorubicin with high accuracy, even if this substance had not been included in the clinical therapy regimens. By contrast, it was not possible to predict *in vitro* drug-specific sensitivity in primary, non-pre-treated human carcinomas with the same degree of accuracy.

To see whether or not our own results reflect the situation observed by other investigators, we performed a survey of all drug sensitivity/resistance results published since 1980 until today. As a first step, we extracted the test results of more than 3600 cancer patients and compared the prediction of sensitivity or resistance *in vitro* with the clinical treatment response (Table 1, *upper part*). The vast majority of publications reported that the prediction of drug resistance was possible with much higher accuracy than prediction of drug sensitivity. In the majority of studies, resistance was correctly predicted with an accuracy between 80 and 100%, while drug sensitivity could only be predicted with an accuracy of only 50–80%. As a next step, we compared our own evaluation of published data with meta-analyses performed by other authors (Table 1, *lower part*). The numbers of patients of all published papers exceeded 15,000. Remarkably again, drug resistance could be predicted with high reliability, but not sensitivity. This synopsis of the published literature of four decades impressively illustrates that the concept of prediction of chemosensitivity was not validated. By contrast, the determination of drug resistance was reliable independent of tumor type, test assay, and drug used in these *in vitro* tests.

**Table 1:** **Predictive value of drug resistance assays**.

Assay	Tumor type	No. of patients	Predictive accuracy	Reference
**Original investigations**				
Tumor clonogenic assay	Ovarian Ca	44	99% resistance prediction; 62% sensitivity prediction	Alberts et al. (17)
^3^H-thymidine and ^3^H-uridine incorporation	Ovarian Ca		84% resistance prediction; 79% sensitivity prediction	Khoo et al. (18)
Extreme drug resistance assay	Ovarian Ca	46	100% resistance prediction; 58% sensitivity prediction	Kern et al. (19)
Fluroescent cytofootprint assay	Ovarian Ca	72	96% resistance prediction; 71% sensitivity prediction	Blackman (20)
Tumor clonogenic assay	Ovarian Ca	93	83% resistance prediction; 50% sensitivity prediction	Federico et al. (21)
ATP luminescence assay	Ovarian Ca	100	>90% resistance prediction (70 untreated, 30 refractory)	Andreotti et al. (22)
Fluroescent cytofootprint assay	Ovarian Ca	47	100% resistance prediction; 56% sensitivity prediction	Csoka et al. (23)
MTT assay	Ovarian Ca	37	85% resistance prediction; 65% sensitivity prediction	Taylor et al. (24)
ATP luminescence assay	Ovarian Ca	38	89% resistance prediction; 66% sensitivity prediction	Konecny et al. (25)
MTT assay	Ovarian Ca	120	83% resistance prediction	Taylor et al. (26)
^3^H-thymidine incorporation	Ovarian Ca	25	100% resistance prediction; 60% sensitivity prediction	Hetland et al. (27)
ATP luminescence assay	Ovarian Ca	61	79% resistance prediction; 60% sensitivity prediction	Neubauer et al. (28)
Sulforhodamine B assay	Peritonitis carcinomatosa of ovarian Ca	28	89.5% resistance prediction; 62.5% sensitivity prediction	Arienti et al. (29)
^3^H-thymidine incorporation	Breast Ca	41	81% resistance prediction; 75% sensitivity prediction	Daidone et al. (30)
Extreme drug resistance	Breast Ca	48	100% resistance prediction; 47% sensitivity prediction	Kern (19)
Fluroescent cytofootprint assay	Breast Ca	47	100% resistance predition; 91% sensitivity prediction	Blackman (20)
ATP luminescence assay	Breast Ca	17	86% resistance prediction; 90% sensitivity prediction	Kochli et al. (31)
^3^H-uridine incorporation	Breast Ca	25	94% resistance prediction; 71% sensitivity prediction	Elledge et al. (32)
MTT assay	Breast Ca	83	80% resistance prediction; 61% sensitivity prediction	Xu et al. (33)
MTT assay	Breast Ca	73	100% resistance prediction; 76.7% sensitivity prediction	Xu et al. (34)
^3^H-thymidine incorporation, tumor clonogenic assay	Gynecological Ca	63	<50% sensitivity prediction; 90% resistance prediction	Eidtmann et al. (35)
^3^H-thymidine incorporation	Gynecological Ca	108	72% resistance prediction; 85% sensitivity prediction	Khoo et al. (36)
MTS assay	Gynecological Ca	45	93.3% resistance prediction; 86.7% sensitivity prediction	O’Toole et al. (37)
ATP luminescence assay	Gastric Ca	36	95.7% resistance prediction; 46.2% sensitivity prediction	Kim et al. (38)
ATP luminescence assay	Gastrointestinal Ca	25	100% resistance prediction; 64% sensitivity prediction	Kawamura et al. (39)
ATP luminescence assay	Esophageal Ca		68.8% resistance prediction; 77.8% sensitivity prediction	Hirai et al. (40)
Tumor clonogenic assay	Liver Ca and liver metastasis	36	71% resistance prediction; 55% sensitivity prediction	Link et al. (41)
Tumor clonogenic assay	Liver Ca	24	91% resistance prediction; 77% sensitivity prediction	Link et al. (42)
Tumor clonogenic assay	Melanoma	50	Retrospective: 100% resistance prediction; 38% sensitivity prediction	Tveit et al. (43)
Tumor clonogenic assay	Melanoma	55	Prospective: 100% resistance prediction; 60% sensitivity prediction	Tveit et al. (43)
ATP luminescence assay	Melanoma	53	83.9% resistance prediction; 36.4% sensitivity prediction	Ugurel et al. (44)
Tumor clonogenic assay	Lung cancer	326	91% resistance prediction; 60% sensitivity prediction	Kitten et al. (45)
Tumor clonogenic assay	Lung cancer	20	86% resistance prediction; 83% sensitivity prediction	Bertelsen et al. (46)
Dye exclusion assay	Lung Ca (SCLC)	21	82% resistance prediction; 55% sensitivity prediction	Gazdar et al. (47)
Collagen gel droplet embedded culture drug sensitvity test (CD-DST)	Lung Cancer (NSCLC)	49	100% resistance prediction; 72.7% sensitivity prediction	Kawamura et al. (48)
Tumor clonogenic assay	Glioma	470	100% resistance prediction; 60% sensitivity prediction	Alonso (1984) (49)
Flow cytometry of DNA integrity	Glioma	41	81% resistance prediction; 86% sensitivity prediction	Iwadate et al. (50)
Dye exlusion assay	Acute leukemia	31	33,3% resistance prediction; 86.7% sensitivity prediction	Hwang et al. (51)
MTT assay	Acute leukemia	31	77,8% resistance prediction; 91.3% sensitivity prediction	Hwang et al. (51)
Tumor clonogenic assay, ^3^H-thymidine incorporation	Multiple myeloma	97	73% sensitivity prediction; 83% resistance prediction	Durie et al. (52)
^3^H-tymidine in corporation	Diverse	33	100% resistance prediction; 46.2% sensitivity prediction	Sondak et al. (53)
^3^H-thymidine incorporation	Diverse	20	93% resistance prediction; 67% sensitivity prediction	Wada et al. (54)
CD-DST	Diverse	554	100% resistance prediction; 80% sensitivity prediction	Kobayashi et al. (55)
Tumor clonogenic assay of xenograft tumors	Diverse	80	62% sensitivity prediction; 97% resistance prediction	Fiebig et al. (56)
ChemoFx^®^ test	Squamous cell Ca, adeno Ca	285	58% resistance prediction; 87% sensitivity prediction	Grigsby et al. (57)
**Reviews**				
Tumor clonogenic assay	Diverse (review)		96% resistance prediction; 62% sensitivity prediction	Salmon et al. (58)
Tumor clonogenic assay	Diverse (review)	>1500	92% resistance prediction; 57% sensitivity prediction	Bertelsen et al. (59)
Tumor clonogenic assay	Diverse (review)		91% resistance prediction; 71% sensitivity prediction	Salmon et al. (60)
Diverse	Glioma (review)		100% resistance prediction; 50-70% sensitivity prediction	Kimmel et al. (61)
Subrenal capsule assay	Diverse (review)	1400	73% resistance prediction; 91% sensitivity prediction	Bodgen and Cobb (62)
Diverse	Review of 54 retrospective studies	2300	91% resistance prediction; 69% sensitivity prediction	von Hoff et al. (63)
Fluorescent cytoprint assay	Diverse (review)		91% resistance prediction; 86% sensitivity prediction	Meitner et al. (64)
Diverse	Diverse (review)	1100	93% resistance prediction; 46.7% sensitivity prediction	Kondo et al. (65)
CD-DST	Diverse	183	88.8% resistance prediction; 79.8% sensitivity prediction	Kobayashi et al. (66)
Tumor clonogenic assay	Diverse (review)	2300	91% resistance prediction; 69% sensitivity prediction	Fiebig et al. (56)
Tumor clonogenic assay	Diverse (review)	66	92% resistance prediction; 62% sensitivity prediction	Fiebig et al. (56)
Diverse assays	Ovarian Ca (review)	1101	93% resistance prediction; 46.6% sensitivity prediction	Kubota and Weisenthal (67)
Diverse assays	Diverse tumor types (review)	4092	90.3% resistance prediction; 71,7% sensitivity prediction	Blumenthal et al. (6)
Diverse	Review of 86 studies	1945	87.4% resistance prediction; 80.0% sensitivity prediction	Weisenthal (68)

Figures 2B,C show that the *in vitro* test results were in good agreement with patients’ survival. Patients, whose tumors were resistant in the *in vitro* short-term test, died earlier than patients, whose tumors were *in vitro* sensitive. This cooperative clinical trial confirmed the feasibility to predict resistance of cancer *in vitro* before starting chemotherapy and the relevance for clinical treatment. In a further study (Figures 2D,E), we investigated surgical adenocarcinoma specimens of the lung (stage III) and compared the *in vitro* short-term test results with survival of patients treated with chemotherapy (16). Thirty-two patients with previously untreated adenocarcinoma of the lung (stage III, pT, pN) were included in this investigation. The minimum follow up time was 5 years. Fourteen patients were only treated by surgical procedures (group S), and 18 patients were additionally treated with cytotoxic drugs (CT group). The chemotherapy protocols used were (a) doxorubicin/cyclophosphamide/vincristine and (b) BCNU/5-fluorouracil, and (c) cisplatinum/vindesine. The survival curves did not differ between the S and CT groups (log-rank *p* = 0.63; rank-sum *p* = 0.39) (Figure 2D). However, if we reanalyzed the same data on the basis of the *in vitro* short-term test results, a different pattern emerged. The CT patients, whose tumors were *in vitro* sensitive lived significantly longer than those with *in vitro* resistant tumors (log-rank *p* = 0.023, rank-sum *p* = 0.006) (Figure 2E). The median survival times were 185 weeks for patients with *in vitro* sensitive tumors and 31 weeks for the patients with *in vitro* resistant tumors. Importantly, there was a statistically significant positive correlation (*r* = 0.7) between the degrees of doxorubicin-induced inhibition of ^3^H-uridine uptake and patients’ survival times. As expected, the survival times of patients treated with surgery, but not chemotherapy, did not correlate with the *in vitro* test results (16). Thus, the observed differences in survival times of patients treated with surgery plus chemotherapy were specific and can be attributed to drug therapy.

Again, we have compared our own data with published results of more than 1900 patients in the literature. As shown in Table 2, patients with tumors that appeared as being sensitive in drug resistance testing had a better survival outcome than patients with drug-resistant tumors. This has been observed in the vast majority of patients independent of which tumors they were suffering from or which cytostatic drug has been used for testing.

**Table 2:** **Prognostic value of drug resistance assays**.

Assay	Tumor type	No. of patients	Prognostic relevance	Reference
MTT assay	Ovarian Ca	120	Survival benefit of sensitive vs. resistant	Taylor et al. (26)
Extreme drug resistance assay	Ovarian Ca	79	Survival benefit of sensitive vs. resistant	Holloway et al. (69)
3D-histoculture assay	Ovarian Ca	164	Survival benefit of sensitive vs. resistant	Nakada et al. (70)
ChemoFx^®^ test	Ovarian Ca	147	Survival benefit of sensitive vs. resistant	Herzog et al. (71)
3D-histoculture assay	Ovarian Ca	104	Survival benefit of sensitive vs. resistant	Jung et al. (72)
MTT assay	Ovarian Ca	120	Survival benefit of sensitive vs. resistant	Xu et al. (73)
3D-histoculture assay	Peritonitis carcinomatosa	18	Survival benefit of sensitive vs. resistant	Isogai et al. (74)
Three-dimensional histoculture	Gastric Ca	128	Survival benefit of sensitive vs. resistant	Kubota et al. (75)
Three-dimensional histoculture	Gastric Ca	32	Survival benefit of sensitive vs. resistant	Furukawa et al. (76)
MTT assay	Gastric Ca	28	Survival benefit of sensitive vs. resistant	Abe et al. (77)
3D-histoculture assay	Gastric Ca	100	No survival benefit of sensitive vs. resistant	Kodera et al. (78)
MTT assay	Gastric Ca	353	No survival benefit of sensitive vs. resistant	Wu et al. (79)
MTT assay	Gastric Ca	50	Survival benefit of sensitive vs. resistant	Kubota et al. (80)
Three-dimensional histoculture	Colorectal cancer	29	Survival benefit of sensitive vs. resistant	Furukawa et al. (76)
MTT assay	Colorectal Ca	200	Survival benefit of sensitive vs. resistant	Kabeshima et al. (81)
MTT assay	Pancreas Ca	14	Survival benefit of sensitive vs. resistant	Yamaue et al. (82)
ATP luminescence assay	Pancreas Ca	18	Sensitive tumors have lower risk of treatment failure than resistant ones	Michalski et al. (83)
MTT assay	Esophageal Ca	46	Survival benefit of sensitive vs. resistant	Nakamori et al. (84)
ATP luminescence assay	Melanoma	53	Survival benefit of sensitive vs. resistant	Ugurel et al. (44)
ATP luminescence assay	Melanoma	14	Survival benefit of sensitive vs. resistant	Doerler et al. (85)
Collagen gel droplet embedded culture drug sensitvity test (CD-DST)	Lung cancer (NSCLC)	49	Survival benefit of sensitive vs. resistant	Kawamura et al. (48)
ATP luminescence assay	Leukemia (ANLL)	23	Survival benefit of sensitive vs. resistant	Möllgård et al. (86)

## Broad Spectrum Resistance

**Figure 3:**
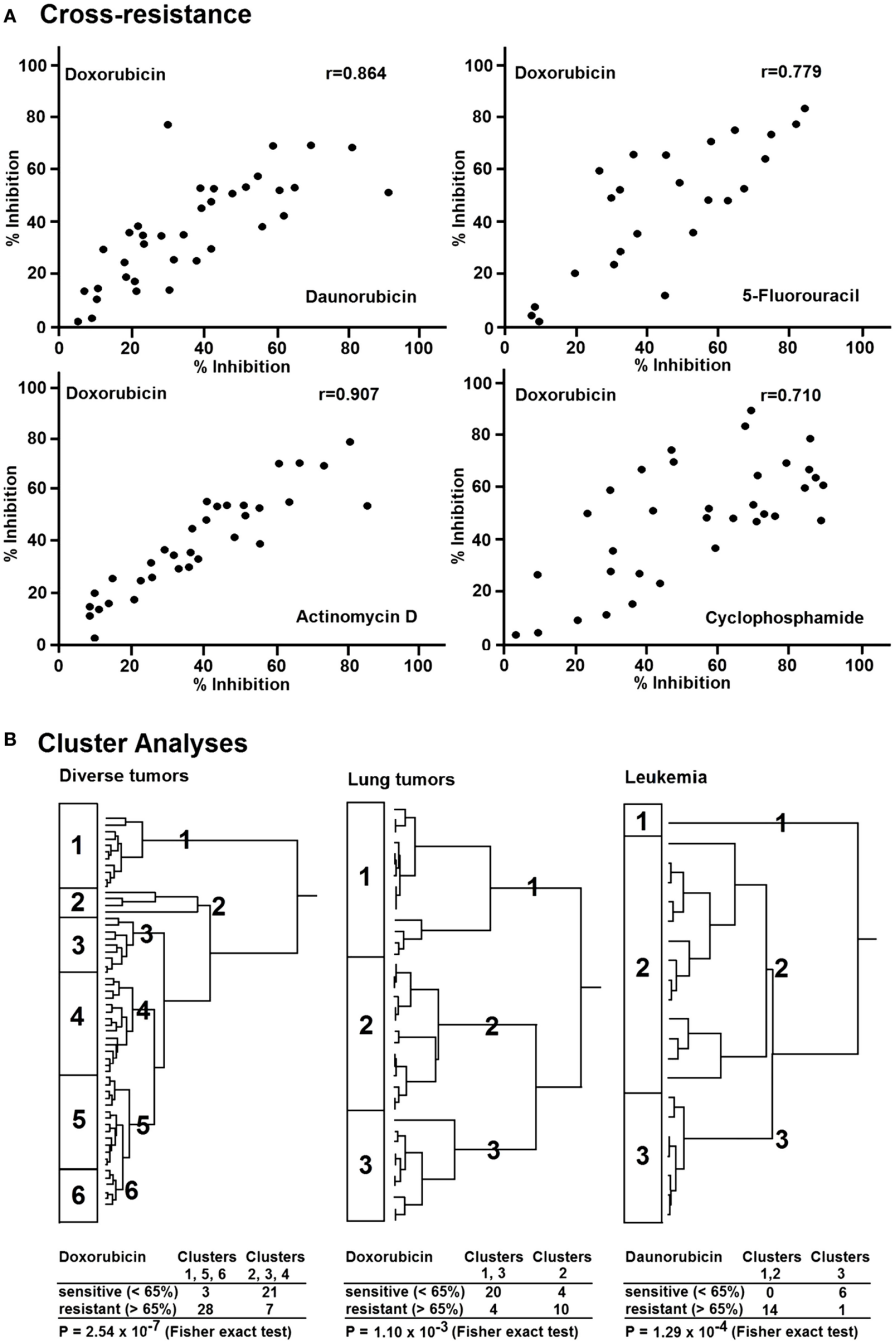
**(A)** Cross-resistance of doxorubicin to daunorubicin, 5-fluorouracil, actinomycin D, and cyclophosphamide in various human tumor types as measured by the *in vitro* short-term test. **(B)** Hierarchical cluster analyses of response of clinical tumor specimens toward different antitumor drugs from different drug classes: doxorubicin, daunorubicin (anthracyclines), actinomycion D, bleomycin (antibiotics), 5-fluorouracil, methotrexate (antimetabolites), mitopodozide (epipodophyllotoxins), and procarbazine, triaziquone (alkylating agents). Dendograms obtained from clustering of 59 diverse tumors, 38 lung carcinomas, and 21 leukemia [data are taken from Ref. (87)].

If a tumor responded to doxorubicin by means of the *in vitro* short-term test, in the majority of cases similar effects can be detected with other cytostatic agents, i.e., tumors insensitive to doxorubicin are also insensitive to other drugs (Figure 3A). Statistically significant correlations existed between the inhibitory effect of doxorubicin, daunorubicin, 5-fluorouracil, actinomycin D, and cyclophosphamide (8). The correlation coefficient for the *in vitro* activities of doxorubicin and daunorubicin was *r* = 0.864 and that for doxorubicin and 5-fluorouracil *r* = 0.779. Similar correlations were also observed between the activities of doxorubicin and actinomycin D (*r* = 0.907) as well as of doxorubicin and cyclophosphamide (*r* = 0.710).

In the 1980s and 1990s, a resistance phenomenon has been investigated termed multidrug resistance (MDR), which is caused by the drug efflux transporter P-glycoprotein. MDR comprises cross-resistance of tumor cells to anthracyclines, *Vinca* alkaloids, taxanes, and epipodophyllotoxins, but not to alkylating agents and antimetabolites (88, 89).

Therefore, we were interested, whether or not the cross-resistance profile observed in our approach was compliant with the MDR phenotype (87). We analyzed 59 tumors of different origin for their *in vitro* resistance to anthracyclines (doxorubicin, daunorubicin), antibiotics (dactinomycin, bleomycin), antimetabolites (5-fluorouracil, methotrexate), epipodophyllotoxins (mitopodozide), and alkylating agents (procarbazine, triaziquone) by means of the *in vitro* short-term test.

As a next step, we performed hierarchical cluster analysis, which may be more suited for an integrated approach to understand the complexity of drug resistance. All investigated drugs except doxorubicin were subjected to cluster analysis (Figure 3B, left). We divided the dendrogram into six clusters and correlated them with the doxorubicin results. Interestingly, sensitive and resistant tumors were separated in the clusters (*p* = 2.5 × 10^−7^). Clusters 1, 5, and 6 (*n* = 31) were enriched with doxorubicin-resistant tumors (54%), whereas clusters 2, 3, and 4 (*n* = 28) were enriched with doxorubicin-sensitive ones. This indicates that resistance to nine compounds from different drug classes (antibiotics, antimetabolites, epipodophyllotoxins, and alkylating agents) significantly correlated with resistance to doxorubicin. This opens the possibility to predict the responsiveness of tumors to a broad range of cytostatic drugs by using solely doxorubicin as a reference drug. To further explore this phenomenon in a group of tumors of the same tumor type, we investigated 38 lung cancers for their response *in vitro* to doxorubicin, 5-fluorouracil, and 4-OOH-cyclophosphamide (Figure 3B, middle). Cluster analysis using the data for 5-fluorouracil and 4-OOH-cyclophosphamide allowed the separation of three clusters. Again, we found a statistically significant relationship between resistance to doxorubicin and resistance to the other drugs (*p* = 1.10 × 10^−3^) in 21 leukemia examples. To validate this result on lung cancer as a solid tumor type, we subsequently used leukemia as single cell cancer. Similar to doxorubicin, daunorubicin as anthracycline of choice for leukemia treatment predicted resistance to other drugs (etoposide, cytosine, arabinoside, 6-thio-guanine) as determined by cluster analysis (*p* = 1.29 × 10^−4^) (Figure 3B, right). In addition to a possible value of doxorubicin as reference compound for the *in vitro* short-term test, another conclusion of these investigations is that tumors exert cross-resistance profiles that are broader than the classical P-glycoprotein-mediated MDR phenotype. Therefore, we have used the term “broad spectrum resistance.” Our point of view on clinical drug resistance phenomena has been recently supported by comparable observations described as “pan-resistance” (90).

Our cluster analyses revealed that clusters of sensitive or resistant tumors could be predicted by one single drug, doxorubicin. This speaks for the high predictive power of doxorubicin to detect broad spectrum resistance. The pleiotropic modes of action of doxorubicin might explain, why doxorubicin is capable of predicting broad spectrum resistance.

1. Doxorubicin is transported by P-glycoprotein and, therefore, is involved in the MDR phenotype. Therefore, doxorubicin can predict response to antibiotics, *Vinca* alkaloids, and epipodophyllotoxins.
2. Doxorubicin inhibits DNA topoisomerase II, which is necessary for cell division. The activity of doxorubicin is dependent on the proliferative activity of tumor cells. Therefore, doxorubicin may predict the responsiveness of tumors to other drugs, which also act in a proliferation-dependent manner, such as antimetabolites, i.e., 5-fluorouracil.
3. The inhibition of DNA topoisomerase II by doxorubicin induces DNA strand breaks. Because alkylating agents also damage DNA, doxorubicin may predict the response to alkylators, such as 4-OOH-cyclophosphamide.
4. Doxorubicin as well as alkylating agents generates reactive oxygen species and radical carbon-centered molecules. This represents another explanation for the predictive power of doxorubicin toward alkylating drugs.

This enables to conduct predictive tumor tests with drugs not included in the therapy schedule. According to our investigations on human tumors, tests using doxorubicin appear to be sufficient to detect drug resistance. Therefore, the *in vitro* short-term test should be generally not used to find the most effective compound in individual tumors, but merely to determine whether a tumor responds at all to any chemotherapy.

## The Multifactorial Nature of Cancer Drug Resistance

Having in mind that tumors tend to be sensitive or resistant not only to single but also to multiple drugs at the same time, it can be speculated that multiple factors rather than single mechanisms may account for broad spectrum or pan-resistance (87, 90). For this reason, we investigated a battery of diverse factors for their expression levels in lung tumors and compared these expressions with the results of the *in vitro* short-term test. The rationale for these analyses was substantiated by the fact that increasing evidence emerged in the literature for a variety of many different drug resistance mechanisms, which are all operative in clinically resistant tumors (5, 13, 88, 91–100). The question arises, as to which resistance factors may be recognized by the *in vitro* short-term test. Therefore, we determined a total of more than 50 resistance-related factors in 94 human non-small cell lung carcinomas by immunohistochemistry (101). These factors can be categorized as resistance proteins, proliferation-related proteins, oncoproteins and tumor suppressor proteins, proteins regulating apoptosis, and angiogenic factors.

The expression of 28 out of >50 proteins significantly correlated with doxorubicin resistance in the *in vitro* short-term test. Of them, the expression of nine proteins directly correlated and another 19 proteins inversely correlated with resistance to doxorubicin.

**Figure 4:**
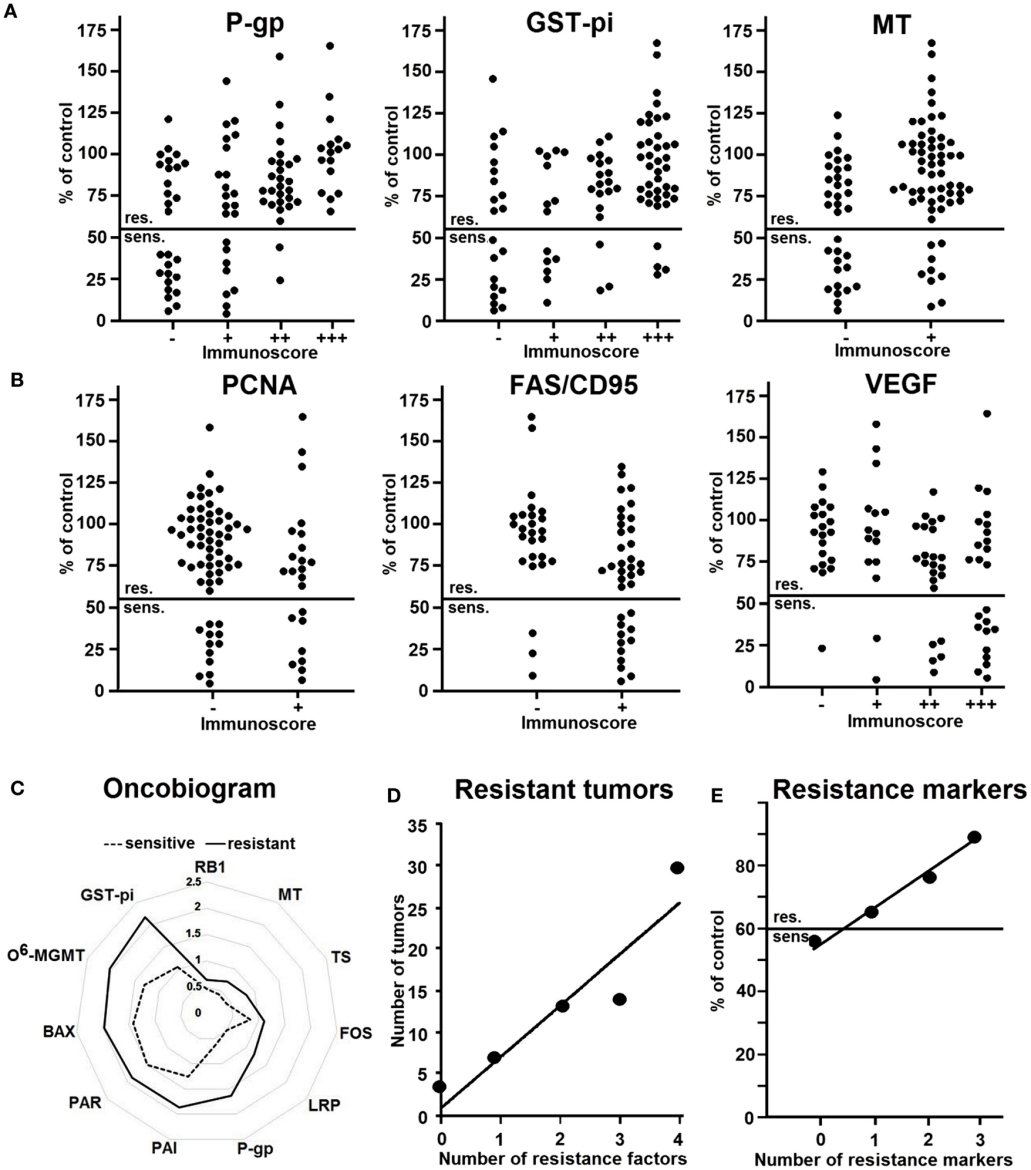
**Relationship between the expression of resistance factors in 94 non-small cell lung carcinomas immunohistochemistry and resistance to doxorubicin as determined by the *in vitro* short-term test**. The factors show no reaction (−) weak (+), moderate (+ +) or strong reaction (+ + +). **(A)** Representative examples of factors directly correlating with resistance. **(B)** Representative examples of factors inversely correlating with resistance. **(C)** Oncobiogram of resistance factors in sensitive tumors (dotted line) and resistant tumors (bold line). **(D)** Number of resistant tumors expressing no or one resistance factor or co-expressing two to four factors (P-gp, GST-pi, TS, MT). **(E)** Number of resistance markers in relationship to the degree of resistance. Abscissa: 0, no resistance marker; 1, one resistance marker, 2, two resistance markers, 3, three resistance markers (P-gp, GST-pi, or TOP2). Ordinate: inhibition by doxorubicin (10 μg/ml) as measured by the *in vitro* short-term test. Abbreviations: P-gp, P-glycoprotein; GST-pi, glutathione S-transferase-pi; MT, metallothionein; PCNA, proliferation cellular nuclear antigen; FAS/CD95, Fas ligand; VEGF, vascular endothelial growth factor; TS, thymidylate synthase; FOS, Fos oncoprotein; LRP, lung resistance protein; RB1, retinoblastoma protein 1; PAI, plasminogen activator inhibitor; PAR, plasminogen activator receptor; BAX, Bcl2 family member; O^6^-MGMT, O^6^-methylguanine DNA-methyltransferase. (Data are taken from Ref. (101)).

Some representative examples are shown in Figures 4A,B. Three examples of resistance proteins that were directly associated with doxorubicin resistance were P-gp, GST-pi, and MT (Figure 4A). These histograms demonstrate that the number of tumors with high protein expression levels (as determined by semi-quantitative immunoscores) increased with doxorubicin resistance.

Figure 4B shows three examples of factors that inversely correlated with resistance, i.e., PCNA, FAS/CD95, and VEGF. Here, rather low than high protein expression was related to doxorubicin resistance. Hence, the number of tumors with high expression of these proteins was higher in sensitive tumors. As a next step, we calculated the mean protein expression values of all sensitive or resistant tumors and plotted them in an oncobiogram. Figure 4C shows a synopsis of all resistance factors that significantly correlated with doxorubicin resistance. It can be clearly seen that the mean expressions of all of these factors were lower in sensitive tumors compared to resistant ones.

These analyses clearly indicate that we have to take multiple rather than single factors into account as mode of action of drug resistance. To prove this assumption, we determined the number of resistant tumors co-expressing more than one resistance factor. Figure 4D shows that the number of resistant tumors expressing four resistance factors was highest, whereas the number of resistant tumors with three, two, one, or no factor was gradually decreasing. This clearly speaks for the multifactorial nature of drug resistance and that single resistance factors are not sufficient to explain resistance phenomena in clinical lung tumors.

In addition, we tested whether combinations of resistance factors may improve the prediction of the degree of resistance. Indeed, the degree of resistance increased with the number of resistance markers (Figure 4E).

## Conclusion

Data obtained from multiple sources, including *in vitro* drug resistance testing, immunohistochemical determination of resistance-related proteins, and clinical data, indicate that no single drug resistance mechanism can explain drug resistance. Resistance mechanisms are numerous and diverse. They depend on the detoxifying capacity of cells, tissue-specific factors, repair capacity, drug delivery, cell proliferation, angiogenesis, apoptosis, and many other factors. Additionally mutation or amplification of specific genes involved in protective pathways as well as the mutation of different oncogene or suppressor genes may be responsible for resistance to chemotherapy. It becomes evident that cancer cells utilize multiple pathways to overcome the cytotoxic effect of drugs used during chemotherapy. Resistance tests should, therefore, recognize these pathways. Our studies attempted to discover the important cellular predictive factors. A key future challenge involves determining the relative contributions of each of these mechanisms.

During the past four decades, various *in vitro* test procedures have been developed used to test sensitivity or resistance. Kubota and Weisenthal reported on *in vitro* and *in vivo* results in 1101 gastrointestinal tumors (67). The correlation of *in vitro* and *in vivo* results revealed 215 true-sensitive (S/S), 246 false-sensitive (S/R), 595 true-resistant (R/R), and 45 false-resistant (R/S), resulting in a 47% true-sensitive rate and a 93% true-resistant rate. Blumenthal and Goldenberg summarized the correlation of the *in vitro* results of different assay types with patients’ response (6). Of 4092 *in vitro* assays, 1809 were sensitive and 2283 resistant. The correlation of *in vitro* and *in vivo* results showed 1297 true-positive patients, who were sensitive *in vitro* and respond to therapy (S/S), 512 false-positive, who were sensitive *in vitro*, but resistant clinically (S/R), 2061 true-negative patients, who were resistant *in vitro* and did not respond to therapy (R/S), and 222 false-negative patients, who were resistant *in vitro* but responded clinically (R/S). The sensitivity was estimated as true in 72% and the resistance in 90% of the cases. Our data are in agreement with all these investigations.

Nevertheless, none of these predictive *in vitro* tests have been clinically established for routine diagnostics. The American Society for Clinical Oncology (ASCO) does not recommend *in vitro* tests for the prediction of chemosensitivity (102, 103). This raises the question as to why clinical translation did not take place, despite numerous investigations speaking for the feasibility of such test systems. An explanation might be the predictive accuracy to detect sensitive and resistant tumors. A close inspection of the data from us and others indicate that independent of the specific test method, drug resistance can be detected with high accuracy (>90%), whereas drug sensitivity can be detected with true-positive rates of only about 40–70%. Hence, the correct conclusion from these data is that all these methods are not reliable enough as clinically useful chemosensitivity tests. However, at the same time it can be stated that drug resistance can be predicted with high reliability. The reasons for this striking difference in predictive power to distinguish between sensitive and resistant tumors may be numerous.

Chemosensitivity of tumor cells detected *ex vivo* under artificial laboratory conditions does not necessarily comply with the specific situation of a patient. For instance, effective levels of antineoplastic agents in tumors may not be reached, if tumors are poorly vascularized. Hepatic biotransformation of drugs or interaction between drugs may also play a role *in vivo*. For these reasons, false-positive results (sensitive *in vitro*, but resistant *in vivo*) can be expected to occur more frequently than *vice versa*. A major concept of all the different predictive *in vitro* tests was to identify drugs *a priori* which tumors are most sensitive to, in order to use them for subsequent therapy. Hence, scientists and oncologists alike were hunting for the optimal chemosensitivity test. The facts after all these years of research teach us that it may not be possible to find such an optimal test system. Therefore, it is time now to rethink and question this concept. Instead of testing chemosensivity, these *in vitro* tests may be used to identify those tumors that are drug resistant with the aim not to treat them with chemotherapy at all. In the past decades, this option may have appeared less attractive, as oncologists cannot leave patients alone with the message “Sorry, your tumor is resistant, we cannot do anything for you.” This is frustrating for both, patients and physicians. Nowadays, the situation is changing, as novel treatment options are emerging. Patients diagnosed as being drug resistant with the help of such predictive tests may be treated with other therapy strategies, such as antibody therapy, adoptive immune therapy, hyperthermia, and in the future may be also with aptamer therapy, gene therapy, and others.

## Conflict of Interest Statement

The authors declare that the research was conducted in the absence of any commercial or financial relationships that could be construed as a potential conflict of interest.
